# From wolves to humans: oral microbiome resistance to transfer across mammalian hosts

**DOI:** 10.1128/mbio.03342-23

**Published:** 2024-02-01

**Authors:** Nicholas A. Podar, Alyssa A. Carrell, Kira A. Cassidy, Dawn M. Klingeman, Zamin Yang, Erin A. Stahler, Douglas W. Smith, Daniel R. Stahler, Mircea Podar

**Affiliations:** 1 School of Engineering, Vanderbilt University, Nashville, Tennessee, USA; 2 Biosciences Department, Oak Ridge National Laboratory, Oak Ridge, Tennessee, USA; 3 Yellowstone Center for Resources, National Park Service, Yellowstone National Park, Wyoming, USA; Harvard University, Cambridge, Massachusetts, USA

**Keywords:** oral microbiome, microbiome evolution, nonhuman microbiota, host-pathogen interactions, host adaptation

## Abstract

The mammalian mouth is colonized by complex microbial communities, adapted to specific niches, and in homeostasis with the host. Individual microbes interact metabolically and rely primarily on nutrients provided by the host, with which they have potentially co-evolved along the mammalian lineages. The oral environment is similar across mammals, but the diversity, specificity, and evolution of community structure in related or interacting mammals are little understood. Here, we compared the oral microbiomes of dogs with those of wild wolves and humans. In dogs, we found an increased microbial diversity relative to wolves, possibly related to the transition to omnivorous nutrition following domestication. This includes a larger diversity of Patescibacteria than previously reported in any other oral microbiota. The oral microbes are most distinct at bacterial species or strain levels, with few if any shared between humans and canids, while the close evolutionary relationship between wolves and dogs is reflected by numerous shared taxa. More taxa are shared at higher taxonomic levels including with humans, supporting their more ancestral common mammalian colonization followed by diversification. Phylogenies of selected oral bacterial lineages do not support stable human-dog microbial transfers but suggest diversification along mammalian lineages (apes and canids). Therefore, despite millennia of cohabitation and close interaction, the host and its native community controls and limits the assimilation of new microbes, even if closely related. Higher resolution metagenomic and microbial physiological studies, covering a larger mammalian diversity, should help understand how oral communities assemble, adapt, and interact with their hosts.

**IMPORTANCE:**

Numerous types of microbes colonize the mouth after birth and play important roles in maintaining oral health. When the microbiota-host homeostasis is perturbed, proliferation of some bacteria leads to diseases such as caries and periodontitis. Unlike the gut microbiome, the diversity of oral microbes across the mammalian evolutionary space is not understood. Our study compared the oral microbiomes of wild wolves, dogs, and apes (humans, chimpanzees, and bonobos), with the aim of identifying if microbes have been potentially exchanged between humans and dogs as a result of domestication and cohabitation. We found little if any evidence for such exchanges. The significance of our research is in finding that the oral microbiota and/or the host limit the acquisition of exogenous microbes, which is important in the context of natural exclusion of potential novel pathogens. We provide a framework for expanded higher-resolution studies across domestic and wild animals to understand resistance/resilience.

## INTRODUCTION

Mammals serve as hosts for microbes that colonize external and internal body surfaces, assembling into distinct commensal communities (microbiota/microbiomes), adapted to specific physiological niches (nutrients, oxygen, and host immunity) (1). Comparative studies of gut microbiomes have revealed common core microbes, primarily at high taxonomic levels (orders and above) across mammals, but divergent in lower-level taxa and community structure, generally tracking host phylogeny and diet (carnivores, herbivores, and omnivores) (2–7). This suggests co-diversification and speciation (8–11), intertwined with responses to differences in diet and environment (3). In birds and bats, on the other hand, flying and the associated physiological and environmental adaptations led to convergent evolution of their gut microbiomes and departure from the typical phylosymbiotic pattern (12, 13).

In mammals, oral and gut microbiome acquisition occurs largely during and shortly after birth, by seeding of the newborn with microbes via maternal and congeneric physical interaction (14–17). During the first weeks and months of extrauterine life, the immune system becomes imprinted with and establishes long-term, dynamic equilibrium with the emerging commensal microbiota. Even though some of the host-microbial specificity could be established at that time, there are ample opportunities for colonization by other microbes from the environment or from other species through cohabitation, food consumption, etc. Stable colonization by species-specific microbes suggests that there may also be genetic determinants of host-microbe, and potentially microbe-microbe interactions that drive community assembly and dynamics (18). Once established, a variety of competitive and antagonistic mechanisms prevent or limit secondary colonization by environmental microbes, including close relatives and pathogens (18–20).

In humans, gut microbial alpha and beta diversity are linked to a plethora of genetic, geographic, ethnic, and dietary factors, with some leading to stable fingerprints and, others fluctuating within individuals and associated with age, health, habits, etc. (11, 21–28). Among the most important factors driving microbiota similarity among individuals are sustained, close interactions and cohabitation, which also appear to support a richer microbial diversity (15, 29–32). Richer, distinct diversity has also been uncovered in native cultures that maintain traditional diets and lifestyles (22, 25–27, 33–35). The pan-microbiome therefore encompasses more microbial taxa and genomic-encoded functionalities than those represented at a given time in any individual or specific population (11, 36–38). Comparatively, the oral microbiota displays fewer short-term variations linked to diet or other individual factors, as there are specific interactions within structured oral biofilms and the bacteria rely mainly on nutrients produced within the oral environment (39–44). During the evolution of the hominid lineages, specific transitions have been documented, even if the core microbiome remained conserved. Most notably, an increased reliance on starch-rich foods in *Homo* lineages that diverged from the Neanderthals led to dominance of *Streptococcus* groups (mitis, sanguinis, and salivarius) that express amylase-binding proteins, facilitating sugar uptake and dental adhesion (45). These transitions indicate that functional and taxonomic changes can be stably incorporated in oral microbiota over relatively short evolutionary time periods, with direct implications to health and disease (e.g., carries and periodontal disease). In addition to nutrition-linked changes, microbiota exchanges occur between humans and companion animals (e.g., dogs) through cohabitation and close physical contact (e.g., many pet owners allow dog face licks including on very young children) (46, 47). Such events are detected based on identifying specific human and dog bacteria. While with potential to cause disease, these bacteria appear to be transient and may not be readily fixed at the family/population levels. Nevertheless, as humans and dogs have cohabited for over 15,000 years since the domestication of grey wolves (48–50), a question arises whether some of the members of their microbiome have not only transiently exchanged hosts but become stable members of the core microbiomes. Domestication and/or captivity are known to lead to compositional and physiological changes in microbiota (4, 51–53). This poses limitations in characterizing the natural diversity and physiology of microbiomes based on sampling animals that live in zoos or other non-native settings. On the other hand, these animal-microbiome systems provide the opportunity to study transient or stable transfer/acquisition of allochthonous microbes across hosts, which is important for understanding microbiome evolution, specificity determinants, and diseases. Here, we aimed to tackle these questions by analyzing human and domestic dog oral microbiomes, under a reference frame that includes wild wolves and non-human primates.

## RESULTS

### Humans and canids have distinctly structured oral microbiota

**Fig 1:**
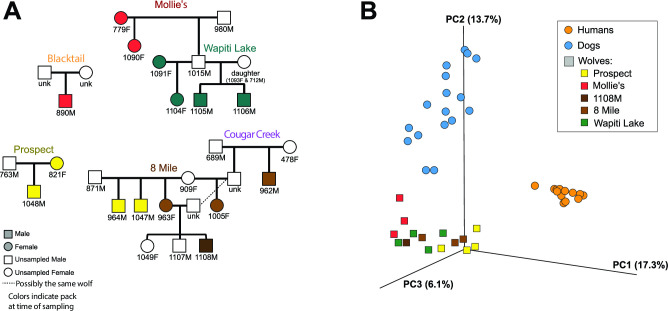
Mammalian hosts and microbial diversity. (**A**) Pedigrees of wolves included in this study with select pack/family relatives. As several wolves left their birth packs and either joined or formed new packs prior to sampling, the colored symbols represent the wolf’s pack at the time of sampling. Only a portion of the pack structure is displayed. (**B**) Beta diversity of oral microbiota (3D principal coordinates Emperor plot using Bray Curtis dissimilarity distances) based on V1–V2 amplicon data. Wolf microbiomes are color-coded based on packs/family.

The mammalian oral environment consists of multiple niches, with microbiota that encompasses both shared and specific microbial taxa (43, 54, 55). In selecting a sampling strategy to be uniformly applied across hosts, we considered the known differences between those niches and the feasibility to rapidly and non-invasively collecting samples in the field. While subgingival communities have high prevalence of strict anaerobes, and in progressing gingivitis and periodontitis those further increase, sampling is somewhat invasive and heterogeneous depending on individual tooth health state and is difficult to perform in the wild. Supragingival plaque, while fully accessible, has few strict anaerobes and is mostly associated with cariogenic microbes. Since oral niches are not physically separate and there is a shared component, to have the highest probability to probe the global oral microbiota, we collected samples from above and immediately below the teeth-gingiva line, which retrieves a mix of sub-, supragingival, and soft tissue communities, as well as small amounts of saliva. For the study, we sampled a wild, pedigreed population of gray wolves (*Canis lupus*) inhabiting Yellowstone National Park (56, 57). Sedated wolves representing five packs (families) (15 individuals) were sampled (Fig. 1; supplemental material). Matching type of samples were also collected from 17 domestic dogs of diverse pure and mixed breeds and from 15 human donors.

**Fig 2:**
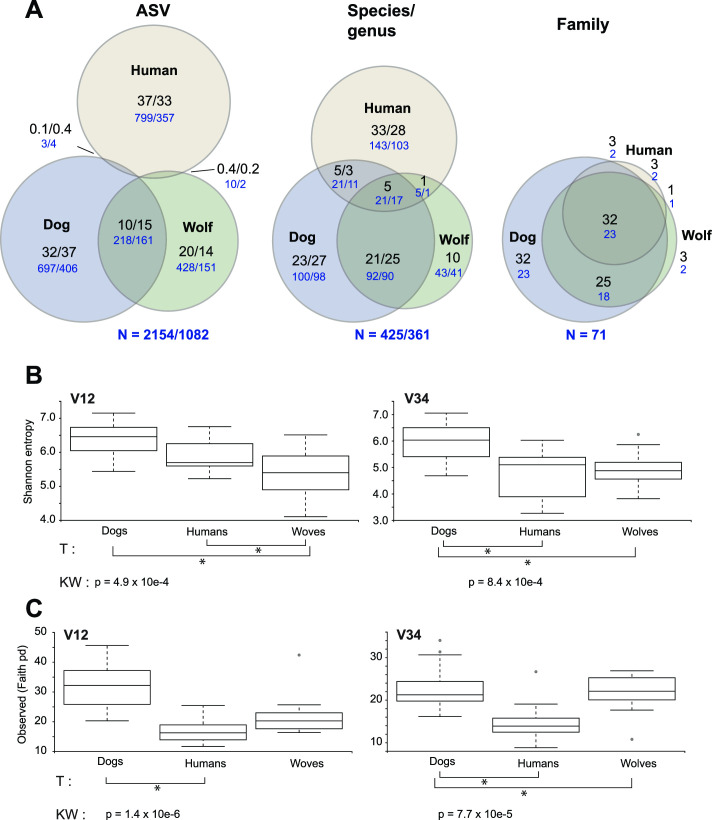
Oral microbiome alpha diversity across hosts. (**A**) Shared and unique microbes at different taxonomic levels (unique amplicon sequence variants, ASVs, species-genera and family). The numbers correspond to actual counts (V1–V2/V3–V4) in blue or represent the percentage of that level (ASV/taxonomic level), in black. N indicates total counts for that level. (**B**) Alpha diversity plots (Shannon and Faith’s diversity) for V1–V2 and V3–V4 between the three hosts. Significant pairwise dissimilarity Tukey’s tests (*P* < 0.05) are indicated with asterisks below the graphs along with Kruskal-Wallis overall diversity dissimilarity tests.

For microbiome diversity characterization and comparisons, we used 16S ribosomal RNA gene amplicons targeting variable regions V1–V2 (mean amplicon length of 316 nt) and V3–V4 (mean amplicon length of 415 nt). While most environmental studies focus on sequencing just V4 or V3–V4, V1–V2 provide increased resolution and coverage for taxa present in animal microbiota (58–61). Indeed, we recovered two times as many amplicon sequence variants (ASVs) for V1–V2 compared to V3–V4 (2,134 vs 1,082) across all samples. Among those, between 14–37% were unique to the individual host species (wolf, dog, and human) and 10–15% were shared between wolves and dogs. Two ASVs assigned to *Clostridiaceae* (likely representing the same bacterium, as those taxa often have some sequence variation between rRNA genes in the same genome) were shared by humans, dogs, and wolves (<0.1%) and 10 or less (<0.5%) were common to human and one of the canids. ASVs can provide resolution below species level for some of the oral microbes and may capture a broader range of evolutionary trends related to microbiota-host co-diversification. After assignment to taxonomic levels (species and genera), while 21–25% of the microbial lineages were shared by dogs and wolves, we also observed 5% common bacteria between all hosts, 3–5% between humans and dogs (Fig. 2), and fewer for human-wolf. When analyzing the family-level assignments, a third of the taxa were shared by all hosts, with the dog presenting a significant number of unique families. Some of those are nevertheless known to be represented in the human oral microbiota but at low levels and primarily at deep subgingival sites affected by periodontal disease (e.g., sulfate reducers and other strict anaerobes).

Alpha diversity analyses revealed distinct community richness between the host species, with dogs presenting the highest Shannon diversity (which accounts for both abundance and evenness) for both amplicons (Kruskal-Wallis all groups test *P* = 4.9–8.4 × 10e^−4^). Similar distinctions were observed when phylogenetic differences between taxa were incorporated (Faith’s phylogenetic diversity) (K-W *P* = 0.1–7.7 × 10e^−5^) (Fig. 2). Other group variables (gender, breed/pack) did not correlate to any measured alpha diversity metrics. The structure of the oral communities was also distinct between the hosts both in terms of overall dissimilarity (Bray-Curtis distances) and phylogenetic composition (UniFrac distances) (Fig. 1; supplemental material) (ADONIS tests *R*^2^ = 0.247, *P* = 0.001 for V1–2, and *R*^2^ = 0.355, *P* = 0.001 for V3–V4 UniFrac distances, respectively). For V1–V2 ASVs, which provide increased taxonomic resolution, there were significant differences in the wolves’ microbiota that were linked to the families/packs (ADONIS test *R*^2^ = 0.41, *P* = 0.02).

### Oral microbiomes combine taxonomic similarities and host-specific characteristics

**Fig 3:**
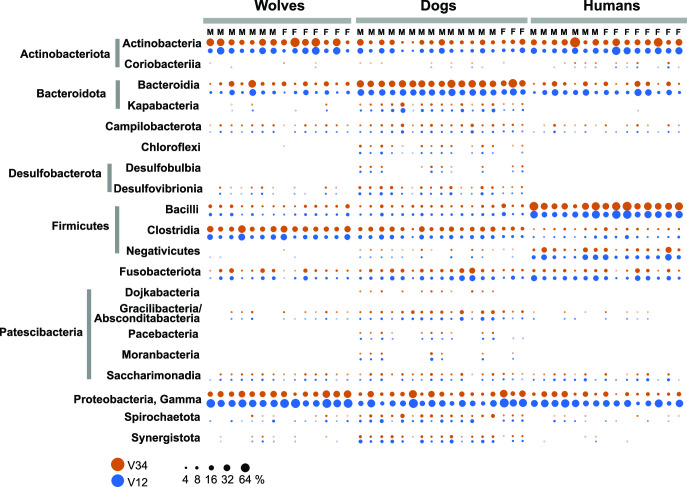
Phylum and class-level microbiome assignments. Bubble plot of relative abundance of bacteria phyla/classes in individual oral microbiomes based on V1–V2 (red) and V3–V4 (blue) amplicon data. Only the top 20 most abundant (>0.1%) taxa are shown. The scale indicates relative abundance in percentage.

Taxonomic analysis of the oral microbiomes revealed an overall composition typical of mammalian supragingival communities, dominated by Actinobacteria, Bacteroidia, Firmicutes (Bacilli, Clostridia, and Mollicutes, at different levels depending on the host), Fusobacteria and Gammaproteobacteria. Overall, the V1–V2- and V3–V4-based results were highly congruent, suggesting a low level of bias linked to ribosomal RNA amplicon type (Fig. 3). Out of the 27 class-level taxa, 15 were shared between all three host species. There were no human-specific microbial classes, and none were uniquely shared between humans and dogs or wolves. Several lineages detected primarily in dogs and/or wolves and in low relative abundance include anaerobes also present in the human subgingival space, especially in periodontal disease (e.g., Methanobacteria, Spirochaetota, Desulfobacterota, Chloroflexi, and Synergistota) (supplemental tables) (62–64). Their greater prevalence in the canide supragingival plaque may be linked to a higher bacterial load compared to humans, which can lead to leaching or expanded anaerobic niches. Nevertheless, three uncultured lineages, as far as we know not previously reported from any mammalian microbiome, were detected in most dogs (but not in humans and wolves), namely Dojkabacteria, Pacebacteria, and Moranbacteria, all members of (super)phylum Patescibacteria. In addition, two other known uncultured oral Patescibacteria groups (Gracilibacteria-GN02, recently merged with Absconditabacteria-SR1) were present at higher relative abundance in dogs than in humans (average 1.8% vs 0.03%), while Saccharimonadia (TM7) were present in all hosts at relatively similar levels (0.4–0.8%) (Fig. 3; Tables S1 to S5). While the dog cohort was not gender balanced, we observed no gender-based differences across and within host species. In humans, large-scale studies identified gender-based differences in the relative abundance of some oral taxa (65).

**Fig 4:**
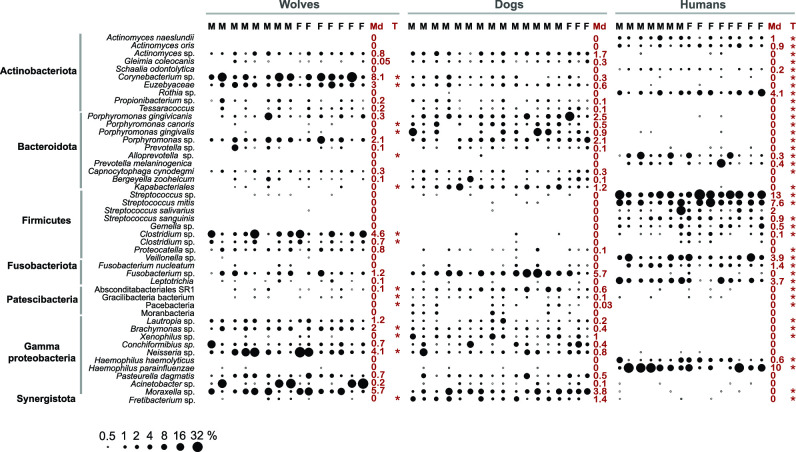
Most relatively abundant species/genera in oral microbiota. Bubble plot based on V1–V2 oral amplicon sequence assignments to species/genera. The scale indicates relative abundance in percentage. F and M indicate the host gender (female and male), Md indicates taxon median relative abundance across the sampled population. Asterisks indicate significant *t* test taxon abundance comparison between dogs and wolves or humans, respectively (*P* < 0.05), after FDR correction.

The main differences between the microbiomes of the three hosts were identified at low taxonomic levels (families and below). As previously known, species’ relative abundance can vary considerably across individuals in oral, gut, and vaginal microbiomes (1, 63, 66). Focusing on the V1–V2 data set, we performed paired *t* tests to evaluate if the relative abundance of individual microbial lineages (>400 species and genera) is significantly different between dogs and humans or wolves, respectively. The ~50 lineages that were found to be significantly more abundant or scarce based on two-tailed *t* statistic (*P* < 0.05) are displayed in Fig. 4, along with their median relative abundances (MRAs). Among the most distinguishable, diverse *Streptococcus* were ubiquitously present in the human population, at MRAs between 1% and 13% but did not exceed 0.1% in the canids (*t* = 7.7, *P* 1.1e−6). *Rothia*, *Gemella*, *Veillonella*, *Haemophilus*, and *Leptotrichia* (MRA 0.5–10%) were also nearly absent in dogs and wolves. For some genera, humans and canids harbored distinct species. In humans, *Actinomyces* was dominated by *A. oris*, *A. naeslundii*, and *A. odontolytica* (*Schaalia*) (MRA 0.2–1%). Those were absent in the canids, which hosted instead a yet-uncharacterized specie (MRA 0.8%). Human- and canid-specific *Fusobacterium* were also detected, at MRAs between 1.4% and 5.7%. When analyzing for specific canid microbiota, a variety of common genera were either not detected or were rare in humans (e.g., *Euzebyaceae*, *Capnocytophaga*, *Brachymonas*, *Neisseria*, *Acinetobacter*, *Moraxella*, with MRAs in dogs and wolves of up to 6%). Diverse species of *Porphyromonas*, some being oral pathobionts (67, 68) and frequent isolates from infected dog bite wounds (69) were highly abundant and ubiquitous in dogs and wolves (exceeding 20% in some individuals). *P. gingivalis*, an important human pathogen that is present in low abundance even in the subgingival microbiota (63, 70), was only detected at low levels (<0.1%) in some humans. Overall, *Porphyromonas* is statistically more prevalent in dogs versus wolves (*t* = 2.4, *P* = 0.01) or humans (*t* = 6.6, *P* = 2.8e-6). Except for Saccharimonadota (MRA 0.14–0.44%), all other Patescibacteria groups (<1% MRA), are strongly associated with dogs based on *t* tests (Pacebacteria *t* = 2.9, *P* = 0.009; Moranbacteria, *t* = 2.2, *P* = 0.03; Gracilibacteria, *t* = 3.0, *P* = 0.006; and Absconditabacteria, *t* = 3.0, *P* = 0.008). Dojkabacteria was identified only using the V3–V4 primers (Fig. 3) (*t* = 3.8, *P* = 0.0009). The only wolf-specific abundant bacteria represented members of the *Clostridiaceae* (MRA 4.8%), occasionally detected at low levels in both dogs and humans (MRA <0.1%) (*t* = 3.6, *P* = 0.002). Additional taxa differentially abundant between the hosts are presented in Fig. 4 along with their MRAs and test significance (Tables S1 to S5 lists all the individual relative abundance data).

**Fig 5:**
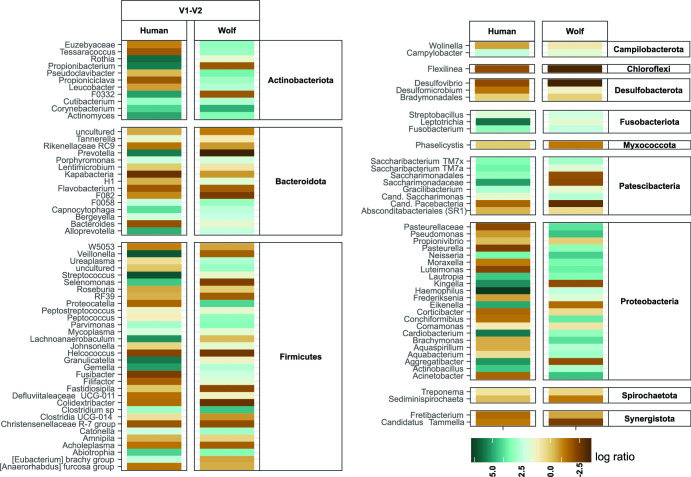
Differential ranking analysis of oral microbiota across mammalian hosts. Log-ratio results are shown for genus level V1–V2 amplicons testing for differences between hosts, with dog as the reference. Colors code for the log-ratio value, with dark brown representing the most negative and dark green as the most positive ratios.

Effective statistical comparisons of amplicon sequence data between samples with unknown microbial load are complicated by their compositional nature (71, 72) and are prone to false positives (73). Therefore, we complemented *t* tests with a differential ranking (DR) analysis approach, which has been shown to be less affected by microbial load (74). Statistical model testing for differences between hosts was conducted by multinomial regression (10,000 iterations) in Songbird (74), for both V1–V2 and V3–V4). Relative differentials (log-ratios of the inferred fold difference in each taxon abundance between two hosts) were calculated (supplemental tables ) and were numerically ranked in Qurro (75) to identify the taxa most different between host pairs (wolves vs dogs and humans vs dogs). They were compared with the *t* test results and converted to a taxonomic-based heatmap (Fig. 5) for easier inspection. Because there were no broad differences between the two data sets, only the V1–V2 log-ratios map is shown. Overall, there is strong agreement between the differentially abundant taxa supported by *t* tests (asterisks in Fig. 4) and DR (dark colors, corresponding to low or high log ratios). As expected, collapsing some of the host-specific species that were negatively correlated between the hosts to genus reduced their log-ratios (*Actinomyces* and *Fusobacterium*). DR pointed to additional, low abundance lineages, as significantly associated with specific hosts in our tested populations. Most of them are facultative anaerobes or uncultured lineages, primarily enriched in dogs. They included members of Bacteroidota (*Cand*. F082, lineages of *Rikenellaceae* and *Paludibacteraceae*, *Flavobacterium*), Firmicutes (*Helcococcus*, *Colidextribacter*, and *Christensenellaceae*), Chloroflexi (*Flexilinea*), Desulfobacterota (*Desulfovibrio*), and Synergistota (*Cand*. Tammella). The Qurro differentials ranking files, provided as supplemental data, enable further in-depth exploration of any specific taxon distribution across the different hosts.

### Phylogenetic testing of potential oral microbial horizontal transfers between hosts

**Fig 6:**
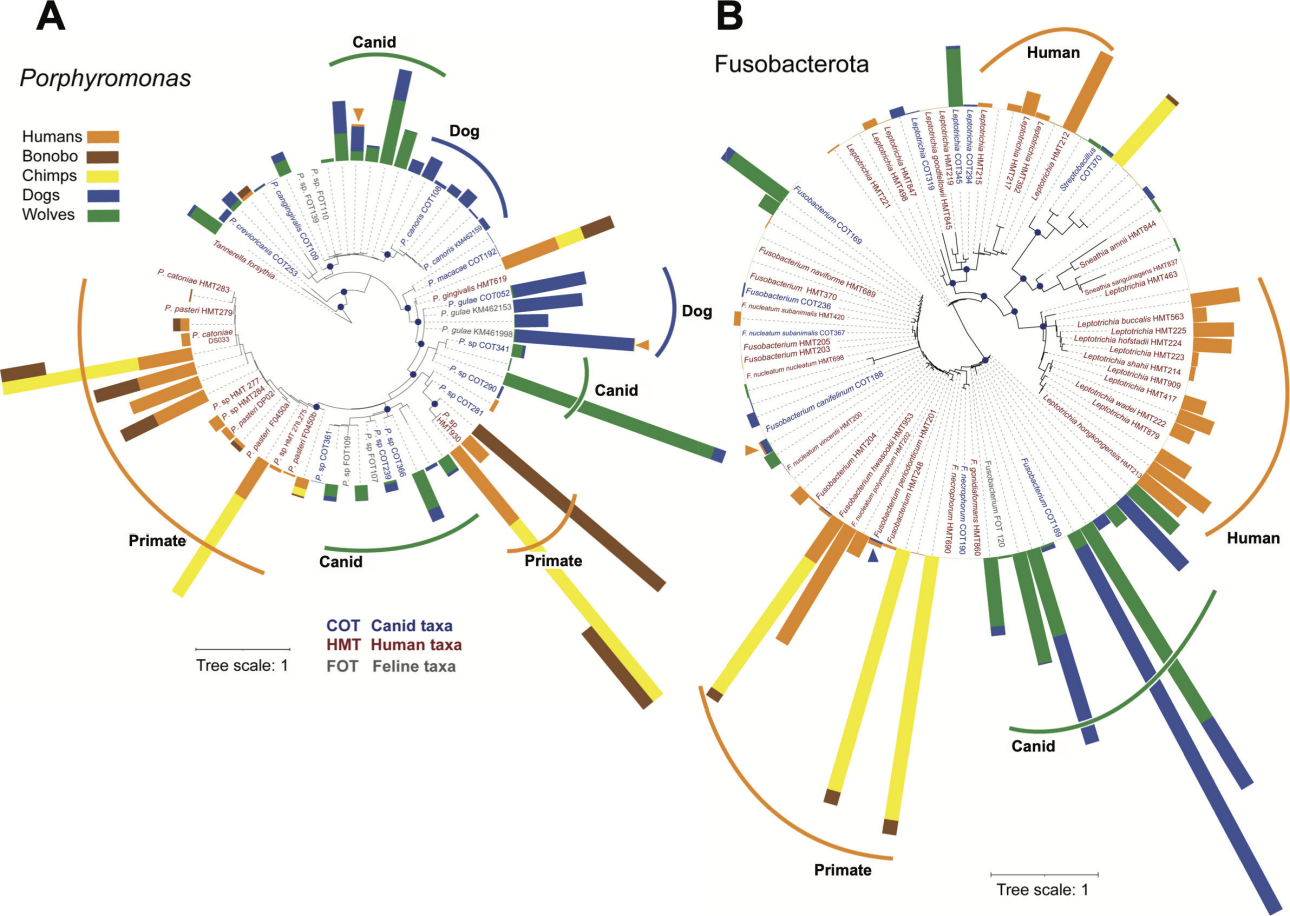
Phylogeny and relative abundance of selected oral microbes. (**A**) Phylogeny of *Porphyromonas* based on rRNA V1–V2 amplicon and sequences from cultured isolates and validated phylotypes (trimmed to V1–V2 region)(rooted with *Tannerella* as outgroup). For each host, the relative abundance of an individual lineage is proportional to the color bars length (summed and normalized to 100% for each host). If few sequences/ASVs matched to any specific lineage/branch, no color bar is visible. Clades that appear specific to various hosts are highlighted. Arrowheads point to potential dog-human transfers. Dark circles at nodes indicate >50% bootstrap support. (**B**) Unrooted phylogeny of Fusobacterota (same description as for panel A).

**Fig 7:**
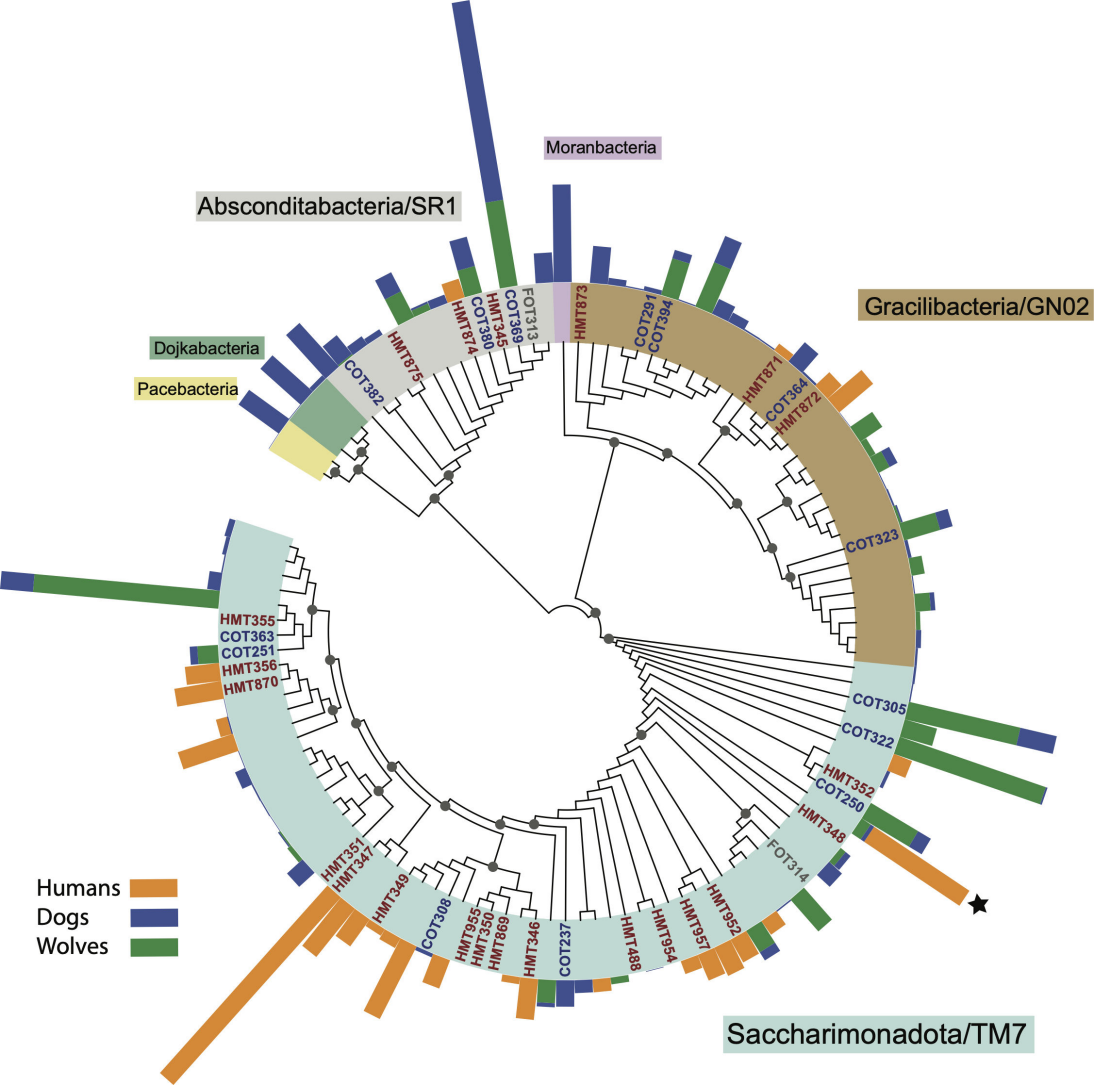
Phylogeny and relative abundance of oral Patescibacteria based on rRNA V3–V4. For each host, the relative abundance of individual lineage is proportional to the color bar length (summed and normalized to 100% for each host). Known lineages (human HMT, dog COT, and feline FOT) are indicated. The colors in the ring outline the individual Patescibacteria lineages on the unrooted cladogram. Dark circles at nodes indicate >50% bootstrap support (only shown for major nodes)

While the taxonomic classification of microbiome sequence data revealed both similarities and differentiating features between the different hosts, they do not distinguish between potential co-diversification with their mammalian lineage and the possibility of lateral transfer. To address that question, we generated phylogenies of representatives from three phyla that have diverse species/strains with distinct distribution across the various hosts (Fig. 6 and 7). We used amplicon sequences obtained here as well as related human and dog rRNA sequences from isolates and independent molecular surveys (76–78). Feline (domestic cat) oral microbial sequences (79) were also included as available. In addition, to trace the evolutionary history of the oral microbes along both primate and canid lineages, we also included sequences from chimpanzees (*Pan troglodytes*) and bonobos (*Pan paniscus*) previously generated by Li et al. (80). Those sequences covered the V–V2 region of the SSU rRNA gene, but because they were generated using pyrosequencing and with different primers, we could not include them in the diversity analyses presented above. In analyzing the phylogenies, we looked for taxa clusters that specifically associated with either primates or canids and for “outliers,” such as human taxa (but not chimp or bonobo) in an otherwise robustly canid (wolf and dog) cluster or vice versa, or dog (but not wolf) taxa in primate clusters. While subject to sampling depth and resolution limitations (imposed by the relatively low evolutionary rates of rRNA gene and the amplicon sequence length), phylogenetic trees could pinpoint if some core human or dog taxa may have a horizontal transfer origin.

One of the most important and diverse disease-associated genera, *Porphyromonas* (Bacteroidota), includes characterized and yet-uncultured species in both humans and dogs. The phylogeny of the V1–2 region of the rRNA gene reveals several relatively abundant canid-specific clusters of species/strains, including two that were only detected in dogs (Fig. 6). Similarly, at least two taxa clusters were specific to primates, the *P. pasteri* group as well as a yet un-named species, *P*. sp HMT930. *P. gingivalis*, a major human periodontal pathogen, and a yet uncultured lineage related to the canid *P. crevioricanis* COT253 appear basal and potentially diverged earlier in mammalian evolution. We identified two distinct human amplicons that were canid-specific, though they were present at low abundance and prevalence. While both were identified in distinct and in more than one individual, because of the low relative abundance we cannot conclude if they represent core constituents of human microbiomes or represent individual-level transfer resulting from recent human-dog interaction. Overall, for *Porphyromonas*, we did not identify any abundant amplicon common to both canids and primates.

Similar host-specific patterns were also observed for Fusobacteria, another group of diverse and highly abundant bacteria in the mammalian oral microbiome (Fig. 6). While some species/strains of *Fusobacterium* (*F. nucleatum*, *F. periodonticum*, and *Fusobacterium* sp. COT189) grouped in primate and canid-specific clades, others could not be effectively resolved and include closely related strains in both humans and dogs (e.g., *F. nucleatum subanimalis* and *F. necrophorum*). As for Porphyromonas, we observed a low level of human amplicon sequences matching canid strain types (*F. cafelinum*) as well as dog sequences matching human taxa (*F. periodonticum*). Again, the very low abundance and distribution of those sequences limits conclusions regarding the origin and acquisition mechanism of those organisms. For another genus of Fusobacteria, *Leptotrichia*, we observed diverse and relatively abundant distribution across human oral samples but, interestingly, those bacteria were not detected in chimp and bonobo samples. Additional primate data will be needed to determine if *Leptotrichia* is indeed much less distributed or even absent among non-human primates. In canids, *Leptotrichia* was detected at relatively low levels and is represented by species/strains distinct from humans.

Another bacterial group that we targeted for phylogenetic characterization is the (super)phylum Patescibacteria, which consists of numerous poorly characterized and largely uncultured lineages, most likely parasitic on other bacteria. Among them, Saccharibacteria has been the most studied and a variety of species/strains have been isolated and characterized from the human oral microbiota and a few other environments. Two others, Absconditabacteria (SR1) and Gracilibacteria (GN02), have few known phylotypes in the human oral microbiota and are also parasites based on genomic data and enrichment cultures. The non-human primate data (chimps and bonobo) did not include any of these bacteria, presumably due to the low sequence coverage of those samples. In dogs, we observed greater relative abundance of Patescibacteria and much higher diversity than in humans (Fig. 7). Wolves shared a variety of dog phylotypes (ASVs) across the three bacterial groups. While some grouping based on host (human vs canids) can be observed, the phylogeny is more complex than for the other bacteria, potentially due not only to the mammalian host but also to co-evolutionary processes between these parasitic bacteria and their specific Actinobacteria hosts. Interestingly, a Saccharimonadia phylotype, most closely related to the human *S*. sp. HMT348, is shared between humans, dogs, and wolves. In addition, so far dogs uniquely host Pacebacteria, Moranbacteria, and Dojkabacteria (Fig. 7). To our knowledge, the first two lineages have not been previously detected in mammalian microbiomes (Dojkabacteria was recently reported in humans [81]) and have no cultured representatives from any environment. When and how they became a part of the mammalian microbiota remains an open question.

## DISCUSSION

Most knowledge on the diversity and evolution of mammalian microbiota has resulted from comparative studies on gut (intestinal) communities, proxied by fecal samples. Gut microbiota is in a dynamic equilibrium, being sustained and constantly replenished as the ingested food transits the intestinal tract. The major microbial lineages that constitute gut microbiota were likely acquired from the environment early in the evolution of mammals, depending on nutrition (e.g., carnivory and herbivory) (e.g., reference 4]). High-resolution phylogenetic and comparative genomic analyses confirmed concerted microbe-host co-diversification across various mammalian lineages, including primates (3, 5, 82–85). Specific environmental or physiological/lifestyle changes in some mammals (e.g., aquatic adaptation, flying, and changing diets) also led to convergent evolution, diversity gain, or loss (2, 4, 5, 7, 12, 85, 86). Horizontal exchange of gut microbes across hosts has also been proposed as a mechanism for microbiota diversification. Such exchange could be mediated through habitat proximity and food sources (87–90). The extent, stability, and evolutionary trajectories of such events are still not well understood.

The dynamics and involvement of oral microbiota in health and a variety of diseases have been studied extensively (55, 64, 91–93). However, in comparison with the gut microbiota, little is known about the diversity and evolution of oral microbiota across mammals, as previous studies focused primarily on domestic animals and other primates (77, 79, 80, 94). A major factor is the difficulty of oral sample collection from wild animals (especially large mammals and carnivores), as compared to opportunistic fecal sampling. The feasibility of oral sampling involves ethical, logistical, procedural, and safety considerations. By swabbing the transition areas on and between teeth and the soft tissues we effectively, rapidly, and noninvasively sampled contiguous oral communities in sedated wolves in their natural setting, an approach that could also be applied to other wild mammals when appropriate and feasible.

Grey wolves were reintroduced in Yellowstone National Park in 1995–1997 and have established dynamic, territorial families (packs) in various areas of the park (57). They have been subject to numerous ecological, behavioral, and genetic studies and are being monitored as part of YNP’s integrated wildlife and environmental conservation and management program (95). Some of the packs’ individuals are periodically tranquilized for mounting tracking collars and for biological data collection (morphometrics, vital parameters, blood, and other samples), the oral microbiota samples analyzed here benefit from that program. A recent independent study analyzed non-oral samples (ear canal, nostril, lip commissure, axilla, dorsal flank, perianal area, and fecal samples) collected from some of the same wolf individuals (56).

The 10-15% shared oral ASVs between domestic dogs and modern grey wolves support their close evolutionary relationship, while the absence of significant canie sharing with humans (much less than 1%) argues against a significant number of stable microbial lateral transfers. The ASVs that are present in both humans and canids belong to *Clostridiaceae*, an occasional human phylotype. Clostridia are primarily intestinal but also present in decaying food. That may explain the higher abundance in wolves, which consume killed prey and scavenged meat and bones, but are also coprophagic (96, 97). Other lineages specific to wolves or shared with dogs include bacteria common in soils or on skin, which may be linked to their social and grooming activities. This highlights the importance of considering environmental and behavioral factors when comparing oral microbiota in various mammals. The correlations we observed between the oral microbial community structure in wolves and their familial (pack) and genetic relatedness agree with those reported by DeCandia et al. (56) on other microbiota communities in YNP wolves. It also indicates that environmental factors do not overshadow the host population and genetics in the assembly and dynamics of the oral microbiota.

The finding that dogs have the highest oral microbiota alpha diversity may be attributable to their secondary adaptation to a more diverse, omnivorous diet, while still preserving some ancestral wolf characteristics. The gut microbiota diversity is also higher in omnivores as compared to carnivores (4). Diverse carbohydrates and more rapidly assimilable nutrients provided in cooked/processed food that dogs have access to are expected to favor the establishment of new microbial guilds, especially in the communities that come in direct contact with the food (saliva, mucosae, and supragingival plaque). An additional factor could also be the high genetic diversity across the many dog breeds that were selected over millennia for various characteristics and tasks (49, 98–100), which may have impacted physiological/metabolic complexity in oral niches. We are not aware of studies that have specifically investigated the oral microbiota relative to breeds, but body size impacts digestion and the gut microbiota in dogs (101), while breed-linked variations have been observed in domestic cats (102). Dietary niche expansion has been associated with microbiota diversification across mammals (3, 82, 103), but there is little comparative information on the oral side. In dogs, a starch-rich diet following domestication led to functional genomic and physiological adaptations (104). In humans and other primates, including fossil lineages, the evolution of the oral microbiota has been associated with changes in environment and nutrition, primarily the utilization of starches and cooked food which drove the increase in diversity of abundance of some genera (e.g., *Streptococcus* and *Veillonella*) (40, 45, 80, 105). Based on our analyses, *Leptotrichia* (a saccharolytic Fusobacterota) has also expanded in humans, with sparse representation in non-human primate data sets. It is possible that the domestic dog diet also led to the expansion of very low abundance ancestral lineages that we could not detect (or were lost) in the sampled wolf population, or their acquisition from other domestic mammals and humans. Because most of the stable oral bacteria have distant or no known free-living relatives and have evolved with hosts along mammalian diversification (e.g., reference 106]), extensive environmental or food-based acquisition appears unlikely. Our phylogenetic analyses support this premise within host diversification for some of the abundant members like *Porphyromonas* and *Fusobacterium*. The case of Patescibacteria is puzzling, as several lineages we consistently detected in dogs have not been reported in other mammalian microbiomes and they are also not common in the environment (soil, plants, and water). Since few mammals’ oral microbiota have been characterized, it will remain to be determined if they could have originated from other domestic animals that dogs may have come into frequent contact with.

Despite the history of co-habitation and frequent physical contact, we did not find indications that any core member of human or dog microbiota has been acquired horizontally. Several prior studies point to potential short-term oral microbiota acquisition events and skin microbiota sharing within the same household (46, 47). Overall, there appear to be significant barriers to colonization, even though the oral environment is quite similar between humans and dogs. An intriguing possibility is that microbiota transfer between hosts could may follow a cross-microbiome route. Some bacteria are present at more than one body site (internal and/or external) and exposure to skin, oral, and fecal communities in cross-species interactions could facilitate such transfers, as it has been shown for gut microbiota (90). In addition, because Patescibacteria may use multiple *bacterial* hosts, speculatively such microbes may be able to transfer more readily across animal hosts. Addressing these questions will require comparisons of a wide range of wild and domesticated animal oral microbiota, ancient oral calculus (107), as well as laboratory studies using isolates. A limitation of our present study is the exclusive use of rRNA amplicons. While they pinpoint oral microbial diversification associated with mammalian lineages, further metagenomic analyses, and targeted isolation and cultivation should provide additional insights and are ongoing.

## MATERIALS AND METHODS

### Wolf sampling

To select a protocol for the collection of oral microbiota samples, logistical, and regulatory constraints on access to wild wolves were a determining factor. Wolves in Yellowstone National Park are sedated by helicopter dart or tranquilized after net gun capture during winter months as part of monitoring, health check, biometric, and genetic studies, under the Yellowstone Wolf Project (https://www.nps.gov/yell/learn/nature/wolf-reports.htm). A short time window is available for data and sample collection on sedated animals and some protocols employed in a human or veterinary dental office (e.g., paper point collection of subgingival fluid samples, site-selective plaque collection using curettes) were not feasible. Therefore, we used DNAGenotek kit for collection of gum and plaque microbiome (OMNIgeneORAL and OMR-110) (DNA Genotek, Ottawa, Canada). After manually exposing the side dentition of the animal, three to four swabs were used to rub the canines, premolars, and molars above and at the gum line (supplemental material), local saliva being collected in that process as well. The swabs were collectively placed in the tube with preservation liquid transported at room temperature and processed as per kit instruction. We collected samples from 15 wolves that were members of five packs, during the 2017–2018 season (supplemental material). Wolf capture, handling, and sample collection protocols were conducted in accordance with the National Park Service (IACUC permit IMR_YELL_Smith_wolves_2012), YNP Scientific Research and Collecting Permit (YELL-SCI-7062 Podar), and ORNL-ACUC (Tracking Protocol 0458, Podar).

### Dog sampling

Domestic dogs free of diagnosed oral disease were sampled by their owners following the same collection procedure used for wolves and in accordance with ORNL-ACUC (Tracking Protocol 0458, Podar). A total of 17 unrelated dogs were sampled, representing 6 pure breeds and 11 various mixed breeds (supplemental material). The dog’s cohort was not gender balanced (we were only able to sample three females), and that could potentially be a source of bias.

### Human sampling

Adult volunteers self-declared to be orally healthy were recruited from the ORNL personnel in accordance with a protocol approved by the Oak Ridge Site-Wide Institutional Review Board (FWA 00005031). Written, informed consent was obtained from all participants (seven males and eight females). The participants self-collected supragingival and gum line plaque from premolars-molars on each mouth side and both jaws in the morning before eating, drinking, or brushing, using OMR-110 swabs.

### Amplicon libraries and sequencing

Total genomic DNA from the collected samples was extracted using a ZymoBIOMICS DNA kit (Zymo Research, Irvine, CA, USA). To prepare SSU rRNA gene amplicon libraries we used the Quick-16S NGS Library Prep Kit (Zymo Research), according to the manufacturer’s protocol for amplification and barcoding. Both sets of included primers (V1–V2 and V3–V4) were used to generate amplicon libraries. Negative controls (no DNA template and blank DNA extractions) were included. Bidirectional sequencing of all samples together was performed on a MiSeq instrument (Illumina, San Diego, CA, USA) using a v3 600 cycles kit, according to the manufacturer’s instructions.

### Sequence processing and diversity analyses

All pairs of raw sequence reads (3.8 million for V1–V2 and 2.8 million for V3–V4), demultiplexed based on samples and primer set on the sequencing instrument, were imported into Qiime2 v.2020.6.0 (108). The negative controls yielded few reads (<100 per control type), indicating no contamination and were excluded. The standard Qiime2 processing workflow was used. Briefly, denoising, trimming (at 18 nt from 5′ end and at 280/240 nt from 3′ end for R1 and R2 reads, respectively), read pairing, removal of chimeras, and identification of ASVs used the dada2 denoise-paired command. For V1–V2, 2.9 million reads were denoised, trimmed, and paired and 2.07 million sequences passed the chimera check (53% of raw reads). For V3–V4, those numbers are 2.1 and 1.2 million (42%), respectively. The total number of ASVs was 4296 for V1–V2 and 2718 for V3–V4. ASVs represented by fewer than 50 sequences totaled across all 47 samples were excluded (2% of all sequences). Between ~12 and 90,000 final sequences were obtained per oral sample for each amplicon type and rarefied to 10,000 sequences for the diversity analyses. Alpha and beta diversity analysis and statistical testing (Kruskal-Wallis *H* test) were performed through the diversity core-metrics-phylogenetic command followed by PERMANOVA tests (999 permutations) for significance based on various metadata (pack, sex, breed). Additionally, Tukey’s HSD of microbial alpha diversity between host species and ADONIS test of the relationship between UniFrac distances and animal species, pack, and/or gender were performed in R, after data import using qiime2R. The ASVs were taxonomically assigned in Qiime2 using trained classifiers derived from the SILVA_138_SSURef_NR99 database (109). Relative abundance data at different taxonomic levels is provided in the supplemental tables. *F* tests were used to determine the variance for each taxon between the hosts (dog vs human and wolf, respectively). To evaluate if relative abundances were significantly different between hosts, we performed paired, two-tailed *t* tests at *P* < 0.05 with FDR correction in R. Bubble plots representing the most relatively abundant and differentially present taxa were generated using a perl script (110). The figures were composed and labeled in Adobe Illustrator.

### Differential ranking analysis

To identify differentially abundant microbial taxa between wolves, dogs, and humans, we applied the reference frames approach implemented in Songbird (74), used it as a Qiime2 plugin and followed the published workflow. Relative differentials were estimated by multinomial regression (10,000 iterations/epochs, differential-prior 0.5, summary-interval 1, and learning-rate 0.0001) using the taxonomy data collapsed to the genus level for both V1–V2 and V3–V4, with host as the reference frame. For differential ranking, model coefficients were checked for fit with visualization of cross-validation and loss as well as validated against null models. For interactive visualization and numerical ranking of the log-ratios, we used the Qurro plugin (75) in Qiime2. The log-ratio values were incorporated in the supplemental tables, and the commands are provided as supplemental data.

### Phylogenetic analyses

ASVs for taxa selected for phylogenetic analyses were imported as fasta files into Geneious Prime v.2021 (111). SSU rRNA sequences from reference oral bacteria (cultured and uncultured) from the Human Oral Microbiome Database (https://www.homd.org/) (112), from dogs and cats (76, 79) were also included. For non-human apes, we included pyrosequenced V1–V2 amplicons from chimpanzees (*Pan troglodytes*) and bonobos (*Pan paniscus*) from two sanctuaries in Africa, reported by Li et al. (80). All sequences were aligned in Geneious using MUSCLE, trimmed to a region common to all input sequences and then manually curated to resolve misalignments followed by masking of positions for which confident alignment could not be achieved. Curated alignments were 196 nt in length for Porphyromonas V1–V2, 220 nt for Fusobacterota V1–V2 and 402 nt for Patescibacteria V3–V4 (we selected V3–V4 because there were no Patescibacteria in the apes V1–V2 data set and only V3–V4 included Dojkabacteria), were used to calculate phylogenetic trees using RAxML version 8.2.11 (113), with GAMMA + P-Invar model of rate heterogeneity, ML estimate of alpha-parameter and 100 rapid bootstrap inferences. The trees were visualized and exported from iTOL (114) after overlaying relative abundance for each taxon calculated for each host species based on the number of mapped sequences.

## Data Availability

The sequence data has been deposited in GenBank SRA under accession PRJNA1040034.

## SUPPLEMENTAL MATERIAL

Supplemental figures mbio.03342-23-s0001.pdf Figures S1-S3.

Supplemental file mbio.03342-23-s0002.txt Analysis commands.

Supplemental tables mbio.03342-23-s0003.xlsx Tables S1-S5.

ASM does not own the copyrights to Supplemental Material that may be linked to, or accessed through, an article. The authors have granted ASM a non-exclusive, world-wide license to publish the Supplemental Material files. Please contact the corresponding author directly for reuse.
