# Supplementary material for: From wolves to humans: oral microbiome resistance to transfer across mammalian hosts
Source: mBio. 2024 Feb 1;15(3):e03342-23. doi: 10.1128/mbio.03342-23 (PMC10936156; doi:10.1128/mbio.03342-23)
Supplement: Supplemental figures — Figures S1-S3. [file mbio.03342-23-s0001.pdf]

964M, Prospect pack

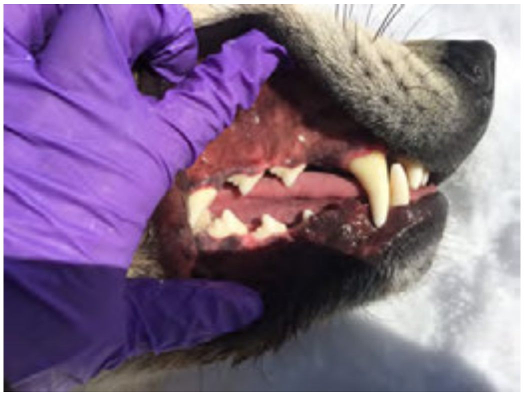

1091F, Wapiti pack

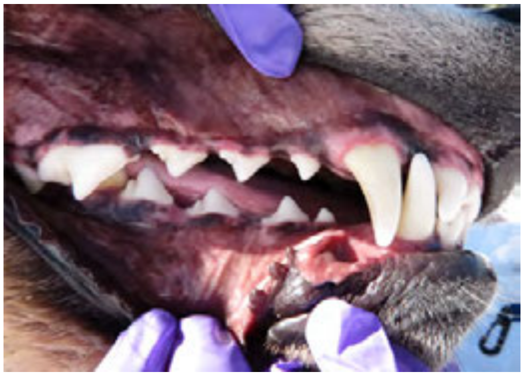

962M, 8 Mile pack

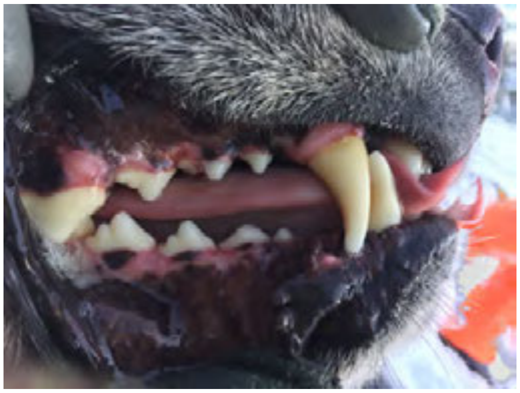

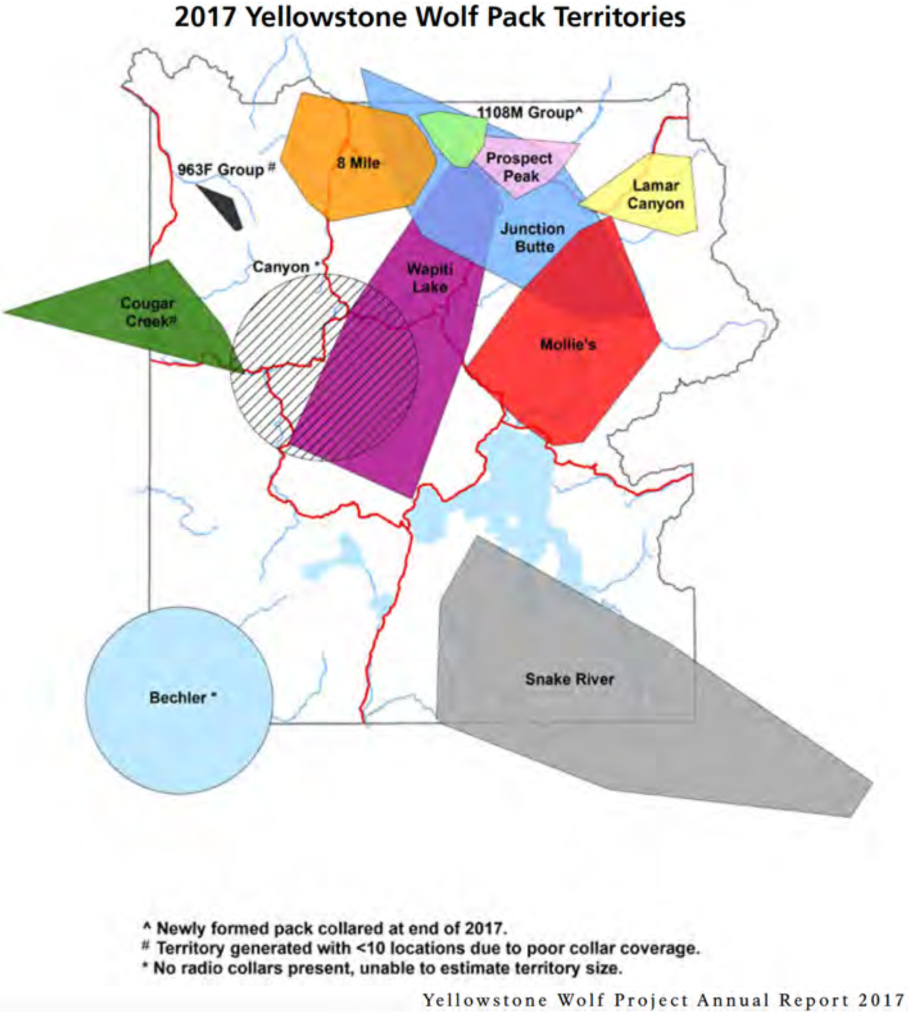

Figure S1. Sample images of wolf dentition prior to sampling. A map with the wolf pack territories in 2017, from the Yellowstone Wolf Project Annual Report 2017, is shown as reference of sampling locations and territories overlap.

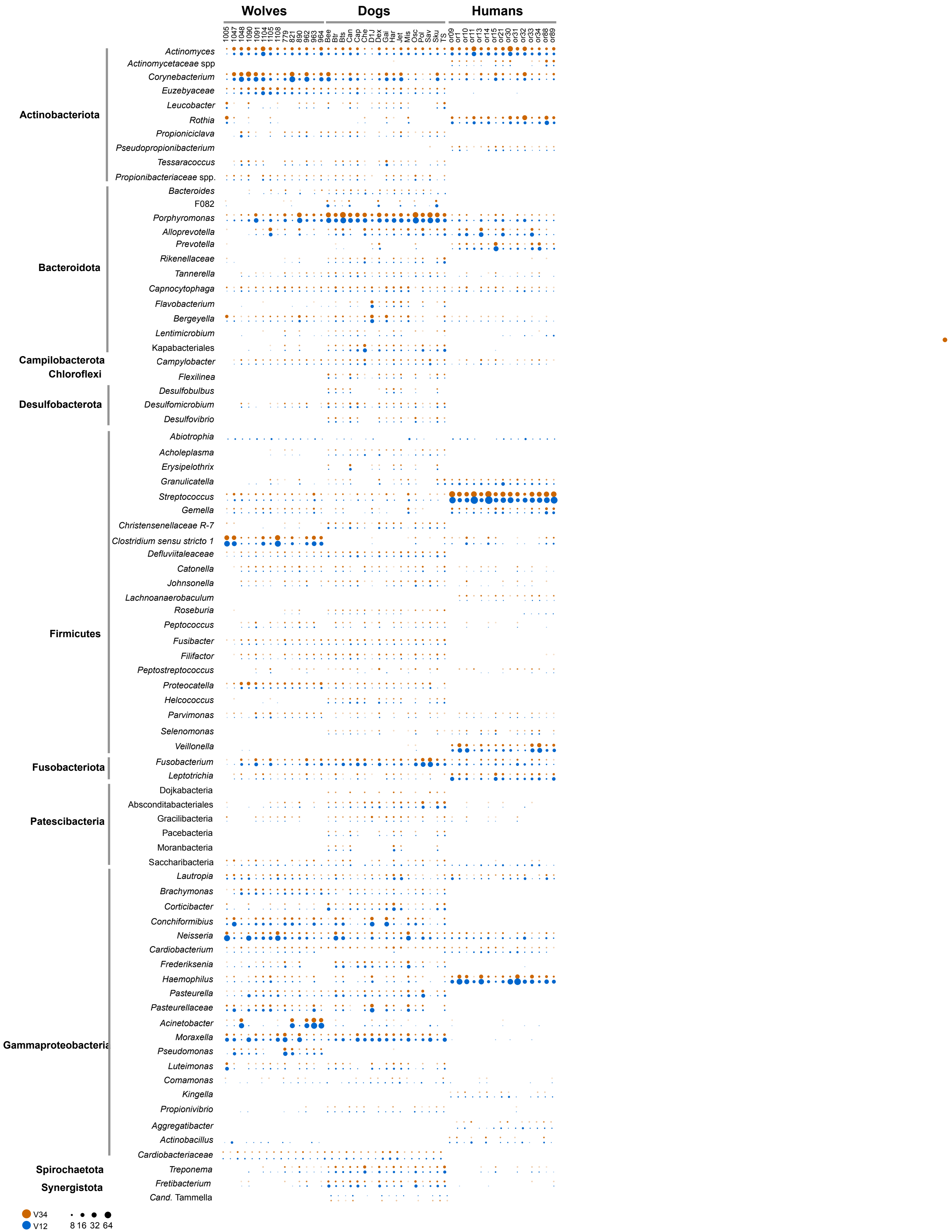

Figure S2. Bubble plot comparison of V12 versus V34 genus level taxonomic assignment for microbial relative abundance across oral microbiomes. The scale is in percentages.

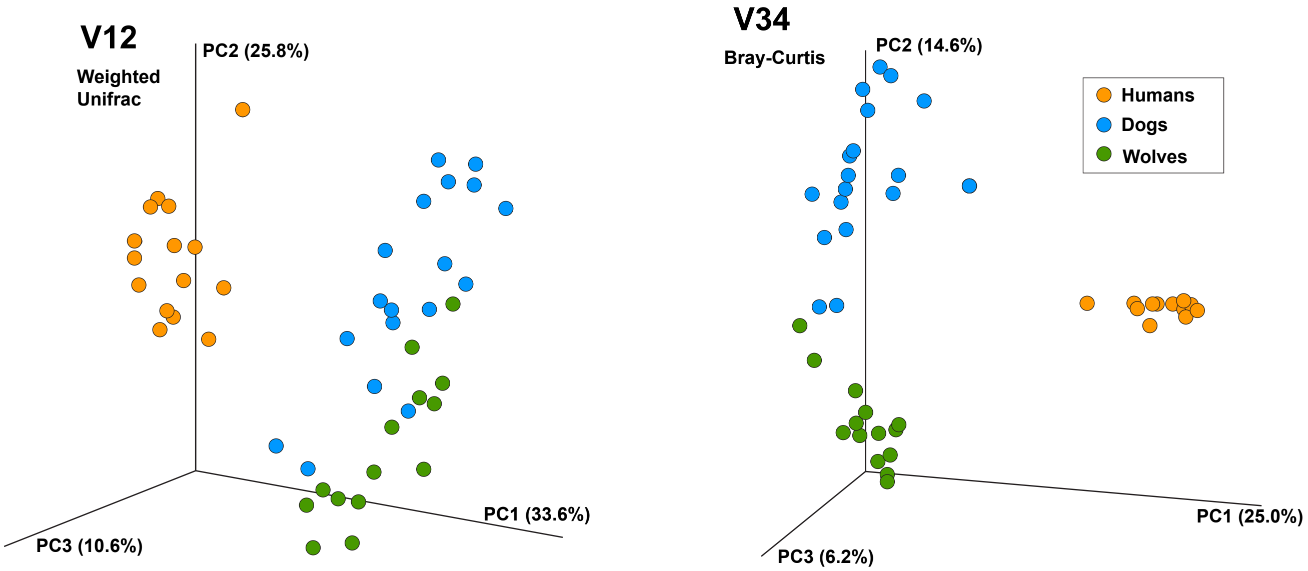

Figure S3. Comparison of beta diversity of oral microbiota (3D principal coordinates Emperor plot) based on weightd unifrac distances for the V12 dataset and for and Bray-Curtis dissimilarity distances for V34.
